# Supplementary material for: Optimization the stochastic optimal reactive power dispatch with renewable energy resources using a modified dandelion algorithm
Source: PLoS One. 2025 Jul 18;20(7):e0328170. doi: 10.1371/journal.pone.0328170 (PMC12273990; doi:10.1371/journal.pone.0328170)
Supplement: Attachment — Submitted filename: Response to Reviewers.docx [file pone.0328170.s002.docx]

**Detailed Response to All Reviewers’ Comments**

| ***Journal:*** | PLOS One |
| --- | --- |
| ***Article number:*** | PONE-D-25-11087 |
| ***Full title:*** | Optimization the Stochastic Optimal Reactive Power Dispatch with renewable energy resources using a Modified Dandelion Algorithm |
| ***Short title:*** | Stochastic Optimal Reactive Power Dispatch |
| ***Authors:*** | Naima Agouzoul, Aziz Oukennou, Faissal Elmariami, Mohamed Ebeed, Jamal Boukherouaa, and Rabiaa Gadal, Mokhtar Aly, Emad A. Mohamed |

**Dear Prof. Emily Chenette**

**Editor-in-Chief, International Journal of PLOS ONE.**

Firstly, the authors appreciate the respected reviewers' interest and invaluable comments. All comments addressed have been carefully considered in the modified manuscript. A separate sheet is attached as a supporting document to clarify modifications highlighted in the previous submission. We would like to have the manuscript considered for publication in this journal.

The authors confirm that this manuscript has not been published elsewhere and is not under consideration by another journal. The authors wish to document the above contributions in the International Journal of PLOS ONE, and we look forward to hearing from you at your earliest convenience.

Sincerely yours,

The authors

**Reviewer #1 comments**

**Comment 1:** “The paper contains multiple language issues that make the text hard to follow (e.g., “increases the complicities,” “application the proposed algorithm”). Careful proofreading is needed to improve readability and clarity. “

**Authors’ Response:** The authors appreciate the reviewer’s feedback regarding language clarity. The manuscript has been carefully revised to correct grammatical errors and improve readability. Problematic phrases such as “increases the complicities” and “application the proposed algorithm” have been addressed.

**Comment 2:** “Some terms and ideas are not clearly defined, like "stochastic fluctuations" and "expected voltage stability." A clearer definition of technical terms would help readers from broader backgrounds.”

**Authors’ Response:** The authors appreciate the reviewer’s comment. The technical terms have been clarified to improve the understanding of the following concepts:

-"Stochastic fluctuations" mean the random variations of the load demand and the power produced by RERs.” To avoid any confusion, we have added “stochastic fluctuations or the random variations “, Kindly refer to line 34 of the manuscript.

-"expected voltage stability “($E{VS}_{scig}$) is a term used to express the evaluation of the ability of power systems to maintain stable voltage levels under power system uncertainties. For a given scenario $scig$^th^, the $E{VS}_{scig}$ is equal to the voltage stability index weighted by the probability of occurrence of the corresponding scenario”. This definition has been added to the manuscript between lines 257 and 260. Additionally, this concept is illustrated in Equation (11) for better clarification.

**Comment 3:** “While the enhancements to the Dandelion Optimizer are interesting, the paper would benefit from a more in-depth discussion of why these specific techniques (QOBL, WFM, FDB) were chosen and how they address limitations of the original Dandelion Optimizer.”

**Authors’ Response:** The authors appreciate the reviewer’s comment. The limitations of the original Dandelion Optimizer have been clarified, along with an explanation of how each proposed method FDB, QOBL, and WFM, addresses these specific limitations. This discussion has been incorporated into the manuscript between lines 162 and 180 through the following newly added paragraph:

*“* In [53] , a comparative study between DO, PSO, GWO and other algorithms was carried out on 22 reference functions in multidimensional experiments. In this analysis, the DO algorithm was compared with PSO [54], which, although showing a good exploitation capacity [55] [56] [57] sometimes suffers from limited exploration and unsatisfactory overall performance . DO was also compared with the GWO algorithm, inspired by the social behaviour of grey wolves. Although GWO showed better performance in exploration, in particular by avoiding premature convergence and maintaining a diversity of solutions in the search space, it nevertheless has limitations in local exploitation, not always allowing precise refinement of solutions [58] [59] [60] .

Despite all the limitations identified for PSO and GWO compared with DO, both algorithms outperformed the latter in terms of exploitation and exploration. The study in [53] ,showed that DO suffer from limited exploration and exploitation capability. To overcome DO's structural limitations, techniques such as Fitness-Distance Balance (FDB) [61], Quasi-Oppositional Based Bearning (QOBL) [62], and Weibull Flight Motion (WFM) [63], were integrated to improve its robustness and efficiency. Specifically, WFM is used to boost the exploration of the applied optimizer, and its effectiveness has been demonstrated in [63] [64] [65]. Similarly, FDB improves global search capability, with successful applications reported [66] [67] [67] [68] [69] [70] [71] [72] .Furthermore, QOBL enhances the exploitation process, as shown in [73] [74] [75] [37] [76] [77] [78].”

It is believed that this addition strengthens the manuscript and provides a deeper understanding of the motivations behind the proposed enhancements.

**Comment 4:** “The contribution of the paper compared to other hybrid optimization approaches needs to be more clearly stated. How does this work advance the state of the art in SORPD beyond existing techniques?”

**Authors’ Response:** The authors appreciate the reviewer’s comment. In response, Table 1 has been added to the manuscript to clearly state the contribution of the proposed approach compared to existing hybrid optimization techniques. Additionally, a brief presentation of the table has been provided between lines 155 and 158.

“The study of existing work has clearly highlighted the advantages and disadvantages of each probabilistic uncertainty handling technics, and it appears that the SBA and MCS methods are more widely used than the PEM. Since our approach is based on the use of scenarios, a specific comparison between MCS and SBA is presented in Table 1. “

Table 1 . Comparison of the proposed method with existing hybrid optimization approaches based on MCS and SBA methods for solving the SORPD problem.

| **Ref** | **Optimization method** | **Year** | **The objective functions** | | **The System** | **Uncertainties** | | | **Number of scenarios** | **Sensitivity Analysis of Scenario Number** |
| --- | --- | --- | --- | --- | --- | --- | --- | --- | --- | --- |
|  |  |  | **power loss**  **minimization** | **voltage stability enhancement** |  | **Solar irradiance** | **Wind speed** | **Load demand** |  |  |
| **Monte Carlo Simulation (MCS)** | | | | | | | | | | |
| [33] | MJAYA | 2021 | ✓ | 🗶 | 30-bus,57-bus | 🗶 | 🗶 | ✓ | 5 | 🗶 |
| [34] | EGWO | 2019 | ✓ | 🗶 | 30-bus | 🗶 | 🗶 | ✓ | 10 | 🗶 |
| [35] | AMRFO | 2023 | ✓ | 🗶 | 30-bus, 118-bus | 🗶 | ✓ | ✓ | 10 | 🗶 |
| [36] | SCENRED2 | 2025 | 🗶 | 🗶 | Sample microgrid | ✓ | ✓ | ✓ | 10 | 🗶 |
| [19] | PSOS-CGSA | 2023 | ✓ | 🗶 | 13-bus | 🗶 | ✓ | ✓ | 10 | 🗶 |
| [20] | EGTO | 2023 | ✓ | ✓ | 30-bus | 🗶 | ✓ | ✓ | 10 | 🗶 |
| [37] | MRUN | 2023 | ✓ | ✓ | 57-bus | ✓ | ✓ | ✓ | 10 | 🗶 |
| [38] | ABWO | 2024 | ✓ | ✓ | 30-bus | ✓ | ✓ | ✓ | 12 | 🗶 |
| [39] | SNOPT-SBB | 2017 | ✓ | ✓ | 30-bus | 🗶 | ✓ | ✓ | 15 | 🗶 |
| [15] | NSGA-II | 2020 | ✓ | 🗶 | 30-bus | ✓ | ✓ | ✓ | 15 | 🗶 |
| [40] | IGWO | 2024 | ✓ | 🗶 | 30-bus | 🗶 | 🗶 | ✓ | 15 | 🗶 |
| [13] | ToP-MOEA | 2023 | ✓ | 🗶 | 30-bus | ✓ | ✓ | ✓ | 20 | 🗶 |
| [41] | SBB | 2015 | ✓ | 🗶 | 30-bus, 118-bus | 🗶 | 🗶 | ✓ | 21 | 🗶 |
| [21] | BiCo | 2023 | ✓ | 🗶 | 30-bus | ✓ | ✓ | 🗶 | 24 | 🗶 |
| [42] | LAPO | 2019 | ✓ | 🗶 | 30-bus | ✓ | ✓ | ✓ | 25 | 🗶 |
| [43] | Rao-3 | 2021 | ✓ | 🗶 | 30-bus, 57-bus, 118-bus | ✓ | ✓ | ✓ | 25 | 🗶 |
| [14] | IMPA | 2022 | ✓ | 🗶 | 30-bus | 🗶 | ✓ | ✓ | 25 | 🗶 |
| [44] | MAHA | 2023 | ✓ | 🗶 | 30-bus | ✓ | ✓ | ✓ | 25 | 🗶 |
| [45] | SHADE-EC | 2019 | ✓ | 🗶 | 30-bus, 57-bus | ✓ | ✓ | ✓ | 25 | 🗶 |
| **Scenario-Based Approach (SBA)** | | | | | | | | | | |
| [46] | SBB -SNOPT | 2016 | ✓ | 🗶 | 30-bus, 118-bus | 🗶 | ✓ | ✓ | 15 | 🗶 |
| [26] | PCA-RCGA | 2020 | ✓ | 🗶 | 30-bus | 🗶 | ✓ | ✓ | 15 | 🗶 |
| [47] | FPSOGSA | 2020 | ✓ | ✓ | 30-bus | 🗶 | ✓ | ✓ | 15 | 🗶 |
| [48] | MSA | 2019 | ✓ | 🗶 | 30-bus | ✓ | ✓ | ✓ | 25 | 🗶 |
| [49] | IMPA | 2022 | ✓ | 🗶 | 30-bus | ✓ | ✓ | ✓ | 25 | 🗶 |
| [50] | ILAPO | 2020 | ✓ | 🗶 | 30-bus | ✓ | ✓ | ✓ | 27 | 🗶 |
| [51] | MPA | 2020 | ✓ | 🗶 | 30-bus | ✓ | ✓ | ✓ | 27 | 🗶 |
| [52] | HFEA | 2020 | ✓ | 🗶 | 14-bus |  | ✓ | ✓ | 75 | 🗶 |
| - | Proposed algorithm | - | ✓ | ✓ | 30-bus | ✓ | ✓ | ✓ | 15 | ✓ |

**Comment 5:** “The paper mentions 15 scenarios for representing load and RER uncertainties but does not clarify how these scenarios were selected or reduced. More insight into the scenario generation and reduction process would add credibility to the stochastic modeling.”

**Authors’ Response:** The authors appreciate the reviewer’s comment. For clarity regarding the scenario generation and reduction process, a brief explanation of the method used has been added between lines 328 and 343, along with appropriate references to relevant literature for readers seeking further details.

“To illustrate the process of generating 800 distinct scenarios using the Monte Carlo Simulation (MCS) method, random values are first generated using the Linear Congruential Generator (LCG). These values are then used as input variables in the inverse functions of the cumulative distribution functions (CDFs) for each probability density function (Weibull, lognormal, and normal). This technique is applied 800 times with independent random draws, generating 800 distinct scenarios. Each of these scenarios is defined as a unique combination of simulated random variables [83] [84].

To optimize computational efficiency, the Scenario-based reduction (SBR) technique was employed, condensing the original 800 scenarios to just 15 representative ones [85] [86] [45]. The SBR follows structured steps that play a role in reducing the number of scenarios from 800 to 15. First, all scenarios are assigned a uniform probability distribution, standing in for the variation in load demand, wind speed, and solar irradiance, adopting equal probabilities. Next, the similarity between the scenarios is assessed by calculating the distance between each pair within the set. Once the closest convergent scenarios have been identified, they are replaced by new scenarios representing their average. This process is then repeated until the required number of scenarios is reached, i.e. 15 scenarios.”

**Comment 6:** “A sensitivity analysis on the number of scenarios would strengthen the argument for the robustness of the proposed approach.”

**Authors’ Response:** The authors appreciate the reviewer’s comment. To address this point, a sensitivity analysis was conducted by varying the number of scenarios (5, 10, and 15) for both the SEPL and SEVS objectives. The corresponding results have been presented in Table 6 and Table 7 of the revised manuscript. Through this analysis, the robustness of the proposed approach with respect to the number of scenarios was confirmed.

Moreover, a short explanatory paragraph was added between lines 699 and 705 to comment on and interpret the obtained results. This analysis confirms the robustness of the proposed approach with respect to variations in the number of scenarios.Haut du formulaire

Bas du formulaire

"The sensitivity analyses of SEPL and SEVS are presented in Tables 6 and 7, respectively. The results are unambiguous and clearly demonstrate robustness. of the proposed MDO approach as the number of scenarios increases. Unlike Algorithms used in this study which experience fluctuations and reduced performance due to variations in the number of scenarios, the MDO systematically achieves the lowest values. This is a clear demonstration of performance stability and reliability. This gradual improvement as scenarios increases highlights MDO's ability to effectively manage complexity and uncertainty, reinforcing its relevance for practical applications.”

**Comment 7:** “The paper should discuss the computational complexity of the MDO algorithm and how it scales with larger systems. Convergence behavior and runtime performance compared to the other optimization techniques should be clearly presented, ideally with convergence plots.

**Authors’ Response:** The authors appreciate the reviewer’s comment. The computational complexity of the MDO has been depicted in the revised paper.

As mentioned before, the DO is based on three stages including rising, landing and descending. Hence, the computational complexity of the DO is $O(M\times T\times pop\times\mathrm{Dim}\times fit)$ where $M$, $T$, $pop$, $\mathrm{Dim}$ and $fit$ are the current optimal solution, the maximum iteration number, number of population, the problem dimension ad the objective function [******** DO]. The MDO is based on three modifications including the WFM, QOBL and FDB, consequently, the computational complexity of the MDO is $O(M\times T\times pop\times\mathrm{Dim}\times fit +3(M\times T\times pop\times\mathrm{Dim}\times fit) )$. Hence, the computational complexity of the MDO is $O(4(M\times T\times pop\times\mathrm{Dim}\times fit))$.

**Comment 8:** “While the results seem promising, the paper would benefit from a more detailed discussion of why the MDO algorithm outperforms others. What specific characteristics of the enhanced MDO contribute to its superior performance?”

**Authors’ Response:** The authors appreciate the reviewer’s comment. As per the results obtained in Table 5, the MDO is superior compared to other algorithms, due to the applied improvement strategies including the WFM, the QOBL and the FDB which boost the searching capabilities of the MDO compared to other techniques.

**Comment 9:** “Statistical analysis of the results (mean, variance, confidence intervals) would provide a more rigorous comparison between optimization techniques.”

**Authors’ Response**: The authors appreciate the reviewer’s comment. the average has already been included in the results; however, "mean" has been replaced with "average" to align with the terminology. Additionally, variance and confidence intervals have been incorporated into the statistical analysis to provide a more comprehensive comparison between the optimization techniques. A short text discussing these statistical results has been added to the manuscript, specifically between lines 711 and 736, along with the results presented in Table 8.

Table 8.Statistical comparison for $\mathbf{SEPL}$, and SEVS for different algorithms in the two case studies for 25 runs.

|  | | | mean | Best | Worst | SD | Variance | 95% confidence intervals | Time(s) |
| --- | --- | --- | --- | --- | --- | --- | --- | --- | --- |
| SEPL | **Without RERs** | SCSO | 98.63769 | 2.30325 | 4.02613e+2 | 97.79830 | 9.564e+3 | [58.25, 139.02] | 7.930e+3 |
|  |  | GTO | 22.35224 | 2.06716 | 2.02490e+2 | 50.01914 | 2.502e+3 | [1.704, 43.00] | 4.471e+3 |
|  |  | BWO | 1.90415e+2 | 2.01590 | 5.02341e+2 | 1.76506e+2 | 3.116e+4 | [117.52, 263.31] | 4.179e+3 |
|  |  | DO | 34.39932 | 2.03062 | 3.02928e+2 | 69.22510 | 4.792e+3 | [5.826, 62.97] | 3.759e+3 |
|  |  | MDO | 18.17287 | **1.87977** | 1.02573e+2 | 37.4685754 | 1.404e+3 | [2.703, 33.64] | 1.576e+4 |
|  | **With RERs** | SCSO | 1.50309e+2 | 1.84510 | 6.02455e+2 | 1.66243e+2 | 2.764e+4 | [81.69, 218.93] | 5.273e+3 |
|  |  | GTO | 22.01844 | 1.60410 | 1.02322e+2 | 40.88172 | 1.671e+3 | [5.13, 38.91] | 3.692e+3 |
|  |  | BWO | 2.66403e+2 | 1.98856 | 7.02699e+2 | 2.34323e+2 | 5.491e+4 | [169.72, 363.09] | 4.046e+3 |
|  |  | DO | 50.03310 | 1.70479 | 3.01924e+2 | 77.05315 | 5.937e+3 | [18.23, 81.83] | 3.674e+3 |
|  |  | MDO | 9.87609 | **1.26277** | 1.02057e+2 | 27.71308 | 7.681e+2 | [0, 21.32] | 1.418e+4 |
| SEVS | **Without RERs** | SCSO | 72.10367 | 0.09962 | 3.00102e+2 | 93.63008 | 8.768e+3 | [33.50, 110.71] | 8.577e+3 |
|  |  | GTO | 88.10130 | 0.09831 | 5.00101e+2 | 1.12989e+2 | 1.276e+4 | [41.47, 134.73] | 4.044e+3 |
|  |  | BWO | 1.521e+2 | 0.09983 | 5.00103e+2 | 1.50332e+2 | 2.260e+4 | [90.10, 213.22] | 4.527e+3 |
|  |  | DO | 52.10132 | 0.09828 | 2.00101e+2 | 65.31979 | 4.267e+3 | [25.18, 78.02] | 3.794e+3 |
|  |  | MDO | 8.10039 | **0.09444** | 1.00100e+2 | 27.68844 | 7.669e+2 | [0, 19.51] | 1.448e+4 |
|  | **Without RERs** | SCSO | 1.76092e+2 | 0.06816 | 7.00080e+2 | 1.80920e+2 | 3.273e+4 | [101.37, 250.81] | 7.320e+3 |
|  |  | GTO | 64.09326 | 0.06811 | 3.00106e+2 | 95.22247 | 9.068e+3 | [24.79, 103.39] | 5.954e+3 |
|  |  | BWO | 2.80102e+2 | 0.09958 | 8.00102e+2 | 2.56579e+2 | 6.586e+4 | [174.20, 386.00] | 9.154e+3 |
|  |  | DO | 52.08767 | 0.06388 | 2.00097e+2 | 71.41761 | 5.104e+3 | [22.61, 81.56] | 7.856e+3 |
|  |  | MDO | 24.08707 | **0.06167** | 1.00096e+2 | 43.58705 | 1.895e+3 | [6.10, 42.08] | 1.685e+4 |

**Comment 10:** “figure 9 is repeated.”

**Authors’ Response:** The authors appreciate the reviewer’s comment. The citation ordering of the Figures has been corrected in the revised paper.

**Comment 11:** “improve figure 1, 9, 10.”

**Authors’ Response**: The authors appreciate the reviewer’s comment. The quality of the mentioned figures has been improved.

- **Reviewer #2 comments**

**Comment 1:** “More details are needed about problem formulation. “

**Authors’ Response:** The authors appreciate the reviewer’s comment. Additional details regarding the problem formulation have been included in the manuscript, specifically in the Problem Formulation section.

**Comment 2:** “Title of 3.2 “Modified Dandelion optimizer. “Title of 4.2 “Modified DANDELION OPTIMIZER “are same.”

**Authors’ Response:** The authors appreciate the reviewer’s comment. The title of section 4. “Modified Dandelion optimizer” has been changed to “DO and MDO modeling” to differentiate it from the title of section 4.2.

**Comment 3:** “Numbers of titles and subtitles should be corrected.”

**Authors’ Response:** The authors appreciate the reviewer’s comment. The numbering of the titles and subtitles has been reviewed and corrected

**Comment 4:** “At figure 12, give a reason, why BWO algorithm is the highest value of SEPL (MW) than other algorithms.

**Authors’ Response:** The authors sincerely appreciate the reviewer’s insightful comment. It is important to emphasize that Figure 12 illustrates the distribution of the objective function, which incorporates both the SEPL and the system constraints. As shown, when applying the BWO method, there is a significant gap between the minimum and maximum values in certain cases, indicating violations of the constraints in trial runs.
