# Supplementary material for: Optimization the stochastic optimal reactive power dispatch with renewable energy resources using a modified dandelion algorithm
Source: PLoS One. 2025 Jul 18;20(7):e0328170. doi: 10.1371/journal.pone.0328170 (PMC12273990; doi:10.1371/journal.pone.0328170)
Supplement: Attachment — Submitted filename: Response to Editor and Reviewers.docx [file pone.0328170.s003.docx]

**Detailed Response to Editor-in-Chief and Reviewers’ Comments**

| ***Journal:*** | PLOS One |
| --- | --- |
| ***Article number:*** | PONE-D-25-11087 |
| ***Full title:*** | Optimization the stochastic optimal reactive power dispatch with renewable energy resources using a modified dandelion algorithm |
| ***Short title:*** | Stochastic Optimal Reactive Power Dispatch |
| ***Authors:*** | Naima Agouzoul, Aziz Oukennou, Faissal Elmariami, Mohamed Ebeed, Jamal Boukherouaa, , Rabiaa Gadal, Mokhtar Aly,and Emad A. Mohamed |

**Dear Prof. Emily Chenette**

**Editor-in-Chief, International Journal of PLOS ONE.**

Firstly, the authors appreciate the respected editor and reviewers' interest and invaluable comments. All comments addressed have been carefully considered in the modified manuscript. The documents titled “**Revised Manuscript with Track Changes**” and the clean “**Manuscript**” are attached to clarify modifications highlighted in the previous submission. We would like to have the manuscript considered for publication in this journal.

The authors confirm that this manuscript has not been published elsewhere and is not under consideration by another journal. The authors wish to document the above contributions in the International Journal of PLOS ONE, and we look forward to hearing from you at your earliest convenience.

Sincerely yours,

The authors

**Editor-in-Chief comments**

**Comment 1:** “Both reviewers identified issues with formatting, language clarity, and technical content.”

**Authors’ Response:** The authors appreciate the editor and reviewers’ comments. A thorough proofreading was carried out. Linguistic and grammatical errors were corrected. Additional explanations have been added to clarify ambiguous terms. The formatting of the manuscript has been revised.

**Comment 2: “**justification for the proposed algorithm modifications “

**Authors’ Response:** The authors appreciate the editor and reviewers’ comments. The limitations of the original Dandelion Optimizer (DO) are that it prone to local optima and suffers from stagnation in case of solving the nonlinear objective functions like ORPD. In this regard, three modifications have been integrated for improving the searching ability of the DO using FDB, QOBL, and WFM.

**Comment 3:** “More rigorous comparison with existing techniques “

**Authors’ Response:** The authors appreciate the editor and reviewers’ comments. A comparsion between the proposed algorithm and the other techniques has included in Table 8 in the revised paper.

**Comment 4:** “detailed analysis of computational complexity “

**Authors’ Response:** The authors appreciate the editor and reviewers’ comments.” It should be mentioned that the proposed MDO has a high level of computational complexity, because the DO is based on three stages, including rising, landing, and descending. Hence, the computational complexity of the DO is O(M×T×pop×Dim×fit), where M, T, pop, Dim, and fit are the current optimal solution, the maximum iteration number, the number of populations, the problem dimension, and the objective function [87]. The MDO is based on three modifications, including the WFM, QOBL, and FDB, Consequently, the computational complexity of the MDO is O(M×T×pop×Dim×fit +3(M×T×pop×Dim×fit)). Hence, the computational complexity of the MDO is O(4(M×T×pop×Dim×fit)).” This explanation is added on the manuscript between lines 508 and 515.

**Comment 5:** “Figure 9 is repeated.”

**Authors’ Response:** The authors appreciate the editor and reviewers’ comments. Figure 9 has been updated to avoid repetition.

**Comment 6:** “improve figure 1, 9, 10.”

**Authors’ Response**: The authors appreciate the editor and reviewers’ comments. The quality of the mentioned figures has been improved on the manuscript.

**Comment 7:** “Please explain the BWO algorithm's higher SEPL value. “

**Authors’ Response:** The authors sincerely appreciate the editor's and reviewers’ insightful comments.

“The BWO algorithm distributes its candidate solutions widely in the search space to avoid premature convergence and accurately explore potential optimal solutions. While this intensive exploration allows local minima to be avoided, it can also lead to less consistent or higher values of the objective function in some runs. It is important to note that Figure 12 shows the distribution of the objective function, which includes both the objective function and the system constraints.
